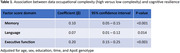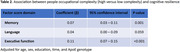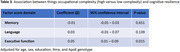# Occupational complexity and cognitive resilience in the Framingham Heart Study

**DOI:** 10.1002/alz.084400

**Published:** 2025-01-09

**Authors:** Phillip H Hwang, Irena Feng, Shruti Durape, Ashita S. Gurnani, Ting Fang Alvin Ang, Sherral A. Devine, Seo‐Eun Choi, Michael L. Lee, Phoebe Scollard, Laura E. Gibbons, Shubhabrata Mukherjee, Emily H. Trittschuh, Richard Sherva, Logan C. Dumitrescu, Timothy J. Hohman, Andrew J. Saykin, Paul K. Crane, Rhoda Au, Lindsay A. Farrer, Jesse Mez

**Affiliations:** ^1^ The Framingham Heart Study, Framingham, MA USA; ^2^ Boston University School of Public Heatlh, Boston, MA USA; ^3^ Boston University Chobanian & Avedisian School of Medicine, Boston, MA USA; ^4^ Department of Neurology, Boston University Chobanian & Avedisian School of Medicine, Boston, MA USA; ^5^ Framingham Heart Study, Boston University Chobanian & Avedisian School of Medicine, Boston, MA USA; ^6^ Boston University Alzheimer’s Disease Research and CTE Centers, Boston University Chobanian & Avedisian School of Medicine, Boston, MA USA; ^7^ Framigham Heart Study ‐ Boston University, Framingham, MA USA; ^8^ Slone Epidemiology Center, Boston University Chobanian & Avedisian School of Medicine, Boston, MA USA; ^9^ Department of Anatomy & Neurobiology, Boston University Chobanian & Avedisian School of Medicine, Boston, MA USA; ^10^ Department of General Internal Medicine, University of Washington School of Medicine, Seattle, WA USA; ^11^ Department of General Internal Medicine, University of Washington, Seattle, WA USA; ^12^ University of Washington, School of Medicine, Seattle, WA USA; ^13^ Geriatric Research, Education, and Clinical Center, Veterans Affairs Puget Sound Health Care System, Seattle, WA USA; ^14^ Department of Medicine (Biomedical Genetics), Boston University Chobanian & Avedisian School of Medicine, Boston, MA USA; ^15^ Vanderbilt Memory and Alzheimer’s Center, Vanderbilt University Medical Center, Nashville, TN USA; ^16^ Vanderbilt Genetics Institute, Vanderbilt University Medical Center, Nashville, TN USA; ^17^ Indiana Alzheimer’s Disease Research Center, Indianapolis, IN USA; ^18^ Department of Medical and Molecular Genetics, Indiana University School of Medicine, Indianapolis, IN USA; ^19^ Indiana Alzheimer’s Disease Research Center, Indiana University School of Medicine, Indianapolis, IN USA; ^20^ Department of Anatomy and Neurobiology, Neurology and Medicine, Framingham Heart Study, BU Alzheimer’s Disease Research Center, Boston University Chobanian & Avedisian School of Medicine, Boston, MA USA; ^21^ Department of Epidemiology, Boston University School of Public Health, Boston, MA USA; ^22^ Departments of Medicine (Biomedical Genetics), Neurology, Ophthalmology, Biostatistics, and Epidemiology, Boston University Schools of Medicine and Public Health, Boston, MA USA; ^23^ Boston University Alzheimer’s Disease Research Center, Boston University Chobanian & Avedisian School of Medicine, Boston, MA USA; ^24^ Department of Biostatistics, Boston University School of Public Health, Boston, MA USA; ^25^ Department of Ophthalmology, Boston University Chobanian & Avedisian School of Medicine, Boston, MA USA; ^26^ Framingham Heart Study, Boston, MA USA; ^27^ Boston University Alzheimer’s Disease Research Center, Boston, MA USA

## Abstract

**Background:**

Greater occupational complexity may be protective against dementia in later life, but it is unclear if it contributes to cognitive resilience and whether different aspects of occupational complexity are associated with resilience. We examined relationships between occupational complexity related to data, people, and things, and cognitive resilience to neurodegeneration.

**Method:**

1,699 participants from the Framingham Heart Study Offspring cohort who were aged ≥60 years, had a plasma total tau (t‐tau) measure (a marker of neurodegeneration), and a neuropsychological (NP) exam visit within five years of the plasma t‐tau measurement were included. Plasma t‐tau was measured using the Simoa assay (Quanterix) on samples collected at Exam 8 (2005‐2008). NP factor scores were previously derived for memory, language, and executive function using confirmatory factor analysis. Occupational data were collected at the NP exam, from which occupational complexity was disaggregated into data complexity, people complexity, and things complexity according to the 1970 US Census Dictionary of Occupational Titles. Cognitive resilience was operationalized using a residual approach by regressing each NP factor score on the plasma t‐tau measure, adjusting for age, sex, education, time from blood draw, and APOE ε4 status. The adjusted residuals were then regressed on each type of occupational complexity, dichotomized into higher complexity versus lower complexity.

**Result:**

The sample was, on average, 70 years of age, 53% female, and had 15 years of education. Higher data (β = 0.20, 95% confidence interval (CI) = 0.15‐0.25, p<0.001), people (β = 0.11, 95% CI = 0.07‐0.15, p<0.001), and things (β = 0.05, 95% CI = 0.01‐0.09, p = 0.015) occupational complexity were most strongly associated with resilience in executive function. Higher data (β = 0.10, 95% CI = 0.05‐0.15, p<0.001) and people (β = 0.07, 95% CI = 0.03‐0.11, p = 0.001) occupational complexity were associated with resilience in memory. Higher data (β = 0.07, 95% CI = 0.01‐0.12, p = 0.014) occupational complexity was associated with resilience in language.

**Conclusion:**

Specific types of occupational complexity contribute to resilience to neurodegeneration in specific cognitive domains differently. Occupational complexity may offer the most resilience in executive function and occupations with high data complexity may offer the most cognitive resilience.